# Combined all‐trans retinoic acid with low‐dose apatinib in treatment of recurrent/metastatic head and neck adenoid cystic carcinoma: A single‐center, secondary analysis of a phase II study

**DOI:** 10.1002/cam4.5653

**Published:** 2023-02-03

**Authors:** Lulu Ye, Lin Zhang, Rongrong Li, Xinhua Pan, Jiang Li, Shengjin Dou, Wen Jiang, Chong Wang, Wantao Chen, Guopei Zhu

**Affiliations:** ^1^ Department of Oral and Maxillofacial‐Head Neck Oncology, Shanghai Ninth People's Hospital, College of Stomatology Shanghai Jiao Tong University School of Medicine Shanghai China; ^2^ National Center for Stomatology Shanghai China; ^3^ National Clinical Research Center for Oral Diseases Shanghai China; ^4^ Shanghai Key Laboratory of Stomatology & Shanghai Research Institute of Stomatology Shanghai China; ^5^ Department of Oral Pathology, Shanghai Ninth People's Hospital, College of Stomatology Shanghai Jiao Tong University School of Medicine Shanghai China

**Keywords:** adenoid cystic carcinoma, all‐trans retinoic acid, apatinib, head and neck cancer, oncological outcomes

## Abstract

**Background:**

Treatment options are limited for recurrent/metastatic adenoid cystic carcinoma of the head and neck (R/M ACCHN). We aimed to evaluate the preliminary results of the efficacy and safety of all‐trans retinoic acid (ATRA) combined with low‐dose apatinib in patients with R/M ACCHN according to a secondary analysis of a phase II study.

**Methods:**

Patients from a phase II study (NCT02775370) who orally administered 500 milligram (mg) apatinib daily until treatment‐related adverse events (AEs) intolerance or progression occurred were eligible for inclusion. Patients were further treated with combination therapy of ATRA (25 mg/m^2^/day) and apatinib (250 mg/day) between March 2019 and October 2021 until progression of disease (PD).

**Results:**

A total of 16 patients were included with nine (56.3%) males and aged 35–69 years old. All recruited patients previously received anti‐angiogenic therapy then withdrew due to toxicities or progression occurred. The objective response rate (ORR) and disease control rate (DCR) were 18.8% and 100%, respectively. During a median follow‐up of 23.9 months (range:17.8–31.7 months), 11 (68.8%) patients developed PD and one of them died in 20.9 months. The median of progression‐free survival (PFS) was 16.3 months (95% CI: 7.2–25.4 months), and the 6‐month, 12‐month, and 24‐month PFS rates were 100%, 81.3%, and 33.3%, respectively. The grade 3 adverse events were albuminuria (*n* = 2, 12.5%) and hand‐foot syndrome (*n* = 1, 6.25%).

**Conclusion:**

All‐trans retinoic acid combined with low‐dose apatinib might be a potential efficacy therapeutic option for patients with R/M ACCHN. This finding will be further confirmed by our registered ongoing trial, the APLUS study (NCT 04433169).

## Graphical abstract

All‐rans retinoic acid combined with low‐dose apatinib might be a potential efficacy therapeutic option for patients with recurrent/metastatic adenoid cystic carcinoma of the head and neck

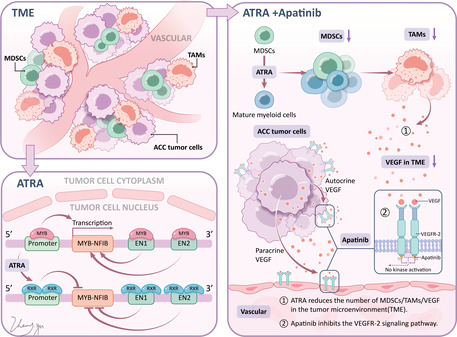

## 1 INTRODUCTION

Adenoid cystic carcinoma (ACC) is a rare subtype of head and neck cancer mostly occurred at salivary glands accounting for 1% of head and neck malignancies.^1^ ACCs usually progress slowly with generally good prognosis.^2^, ^3^ However, patients treated with surgical resection often develop local and repeated recurrence,^4^ and nearly half of patients develop distant metastases, and up to one third die within 2 years of diagnosis.^5^, ^6^, ^7^ Neither chemotherapies nor trials of targeted therapy agents up‐to‐date have shown sufficient efficacy, therefore effective therapeutic regimen for relapsed patients of ACC is still lacking.^8^ Since angiogenesis in tumor has been suggested to play a critical role in ACC pathogenesis,^9^ agents targeting vascular endothelial growth factor (VEGF) signaling has been trialed for ACC treatment. Apatinib is a new generation of highly specific small molecule VEGFR‐2 tyrosine kinase inhibitor. The main mechanism of action for apatinib is preventing VEGFR‐2 downstream signaling pathways and blocking the migration and proliferation of vascular endothelial cells, reducing tumor microvessel density, and inhibiting tumor angiogenesis.^10^, ^11^, ^12^, ^13^ Based on the phase I and phase II clinical trials, the China Food and Drug Administration (CFDA) approved apatinib as the third‐line treatment for advanced gastric cancer or adenocarcinoma of the gastroesophageal junction in 2014. We previously conducted a phase II trial to evaluate the efficacy of apatinib on recurrent/metastatic head and neck ACC (R/M ACCHN) (ClinialTrials.gov NCT02775370) and showed that the 6‐, 12‐, and 24‐month progression‐free survival (PFS) rates were 92.3%, 75.2%, and 44.7%, respectively, in 68 patients who received 500 milligrams per day (mg/d) of continuous apatinib therapy. Moreover, patients who received a higher intensity of apatinib treatment over 6 months had significantly longer PFS than those who received a lower intensity of apatinib. Nevertheless, 50 (73.5%) patients required dose reduction due to intolerance and adverse events (AEs).^14^ Poor tolerance of monotherapy apatinib with 500 mg/d suggested the treatment needs to be further optimized.

All‐trans retinoic acid (ATRA), a major metabolic derivative of vitamin A, is a critical lipid signaling molecule in regulating embryonic and postnatal development. As an inducer of tumor cell differentiation,^15^ ATRA is a potential anti‐tumor drug. The agent has been approved for clinical treatment of leukemia and tested in off‐label usage and clinical trials for various solid tumors.^16^, ^17^ In addition, a positive feedback loop of MYB signaling pathway suggested to be involved in ACC pathogenesis and MYB targeted treatment has been attempted in ACC.^18^, ^19^ Recently, it is reported that ATRA could inhibit the expression of MYB and dramatically lower the level of the oncogenic fusion protein, suggesting a potential treatment agent for R/M ACC.^20^, ^21^ However, less efficacy with 3.2 months mPFS (95% CI, 1.8–3.9) of ATRA alone in advanced ACC patients was previously reported in a phase II trial (ClinialTrials.gov NCT03999684), and ATRA in combination may be a low toxicity choice for disease growth stabilization in R/M ACC.^22^

As an indolent tumor, ACCs were characterized by an immune‐excluded microenvironment, the presence of M2‐polarized macrophages and myeloid‐derived suppressor cells (MDSC), and very low mutational load are the key drivers of resistance to antiangiogenic therapies.^23^ ATRA induces differentiation of MDSC into mature cells and it is reported that ATRA alone in combination with chemotherapy could improve the therapeutic effect of antiangiogenic therapies in many solid cancers.^24^, ^25^

**FIGURE 1:**
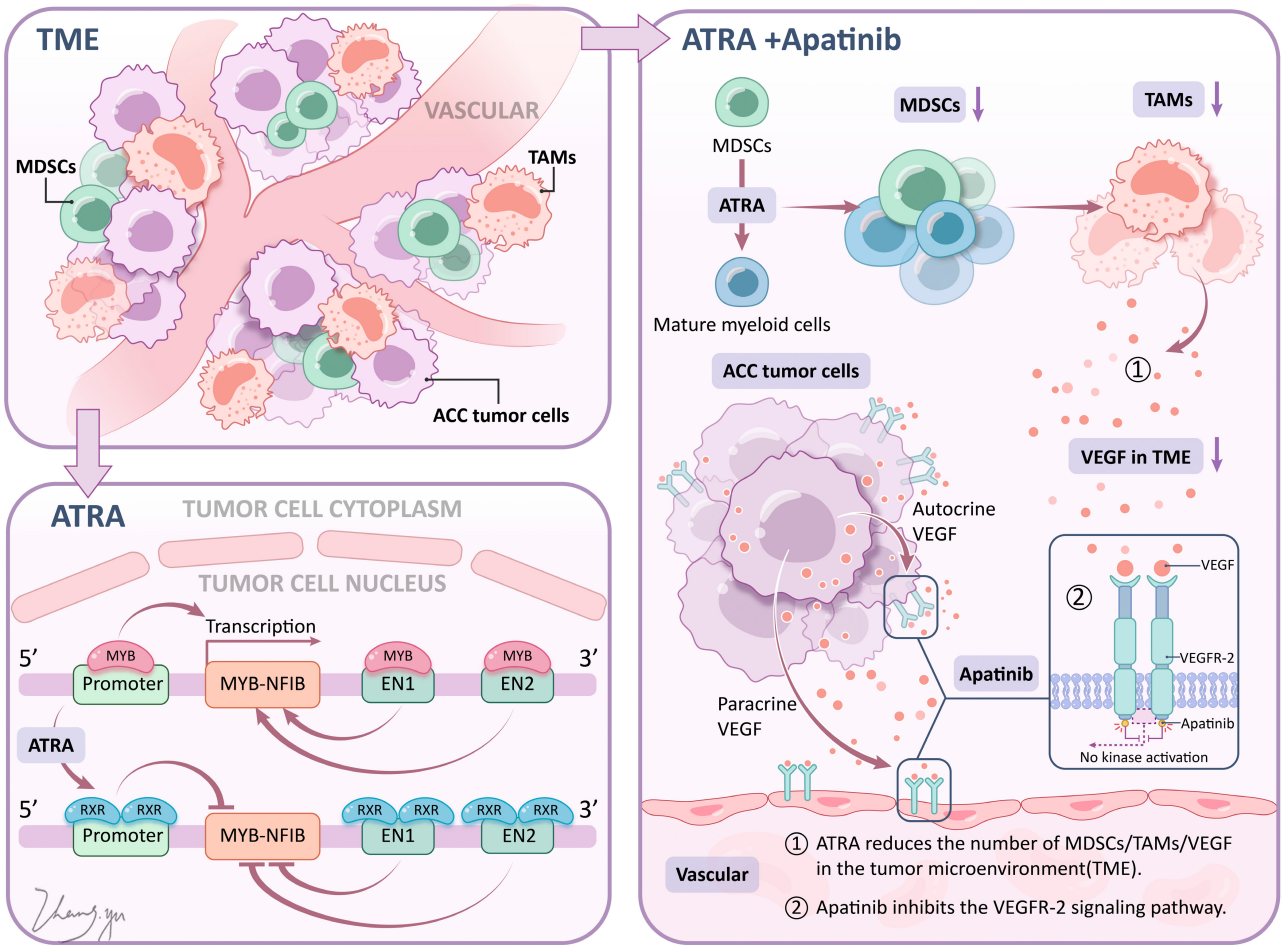
The potential mechanism of action of ATRA/Apatinib combination.

Less efficacy of monotherapy ATRA and poor tolerance of monotherapy apatinib limited their usage in clinical practice, and the combination of ATRA and Low‐dose apatinib may be a promising new approach. As we know, the two positive feedback loops mediated by MYB and VEGF in ACC, and in mechanism, combination of ATRA and apatinib could block these two loops at same time (Figure 1). ATRA diminish the MYB positive feedback loop driving ACC.^20^ Apatinib is an inhibitor of the VEGFR‐2 receptor,^11^ and ATRA can induce MDSC maturation, which reduces MDSC, thereby reducing tumor‐associated macrophages (TAM) and VEGF secreted by TAM. The combination of apatinib and ATRA could synergistically inhibit the positive feedback loop of VEGF.^24^ In order to evaluate whether combined therapy of ATRA and low‐dose apatinib could improve the efficacy and reduce the side effects of apatinib, we further secondary analyzed 16 patients of R/M ACCHN in a phase II study of prior received monotherapy apatinib. These patients withdrew drug due to treatment‐related AEs and were exploratory accepted combination therapy of ATRA (25 mg/m^2^/day) and low‐dose apatinib (250 mg/day).

## 2 MATERIALS AND METHODS

This study was a secondary analysis of a phase II study which was approved by the ethics committee of our hospital (SH9H‐2020‐T73‐1). The design of study and the inclusion and exclusion criteria were detailed in previous publication.^14^

All patients included in this study had experienced dose reduction in the phase II study, and have discontinued apatinib for 6 months or more before accepting the treatment of this study. The inclusion criteria were (1) >18 years of age; (2) advanced or R/M ACCHN diagnosed by pathology or histology; (3) with measurable lesions by spiral computed tomography (CT)/ magnetic resonance imaging (MRI) scan ≥10 mm and meeting the RECIST 1.1 criteria; (4) apatinib was used in the last‐line therapy and discontinued for AEs or progression; (5) Eastern Cooperative Oncology Group (ECOG) performance status (PS) 0–1, and (6) treated with ATRA 25 mg/m^2^/day and apatinib 250 mg/day; (7) all patients provided written informed consent before inclusion.

The exclusion criteria were (1) previous or coexisting malignancies (except cured basal cell carcinoma of the skin, and carcinoma in situ of the cervix) or (2) incomplete data, such as missing outcome indicators.

### 2.1 Efficacy outcomes and safety measures

The primary study endpoint was PFS, according to RECIST version 1.1, which is defined as the time from enrollment until objective tumor progression or death. OS is defined as the time from patient enrolment to the time of death from any cause. The partial response (PR) is defined as at least a 30% decrease in the sum of the target lesions. Stable disease (SD) is defined as fitting the criteria neither for progressive disease nor a PR. Objective response rate (ORR) is the percentage of patients with partial response and/or complete response. Disease control rate (DCR) is the percentage of patients achieved complete response, partial response, and stable disease. Patient characteristics (age, sex, lesions, treatment lines, and the number of ATRA/apatinib cycles) and treatment outcomes (best response, PFS, complications, AE and follow‐up) were extracted from the medical charts and telephone follow‐up. Safety evaluations at all study visits included laboratory and adverse event (AE) assessments adhering to NCI Common Terminology Criteria version 4.0. The patients were evaluated every two cycles. The last follow‐up time was in October 2021.

### 2.2 Statistical analysis

SPSS 22.0 (IBM, Armonk, NY, USA) was used for statistical analysis. Continuous data were tested with the Kolmogorov–Smirnov test for normal distribution. Normally distributed continuous data are expressed as means ± standard deviations, and non‐normally distributed continuous data are expressed as median (ranges). Partial response (PR), stable disease (SD), progression of disease (PD), objective response rate (ORR), disease control rate (DCR), overall survival (OS), and PFS were calculated by using the exact binomial distribution. OS and PFS were estimated by using the Kaplan–Meier method. Only descriptive statistics were used and none of the statistical tests were applied.

## 3 RESULTS

### 3.1 Characteristics of the patients

**FIGURE 2:**
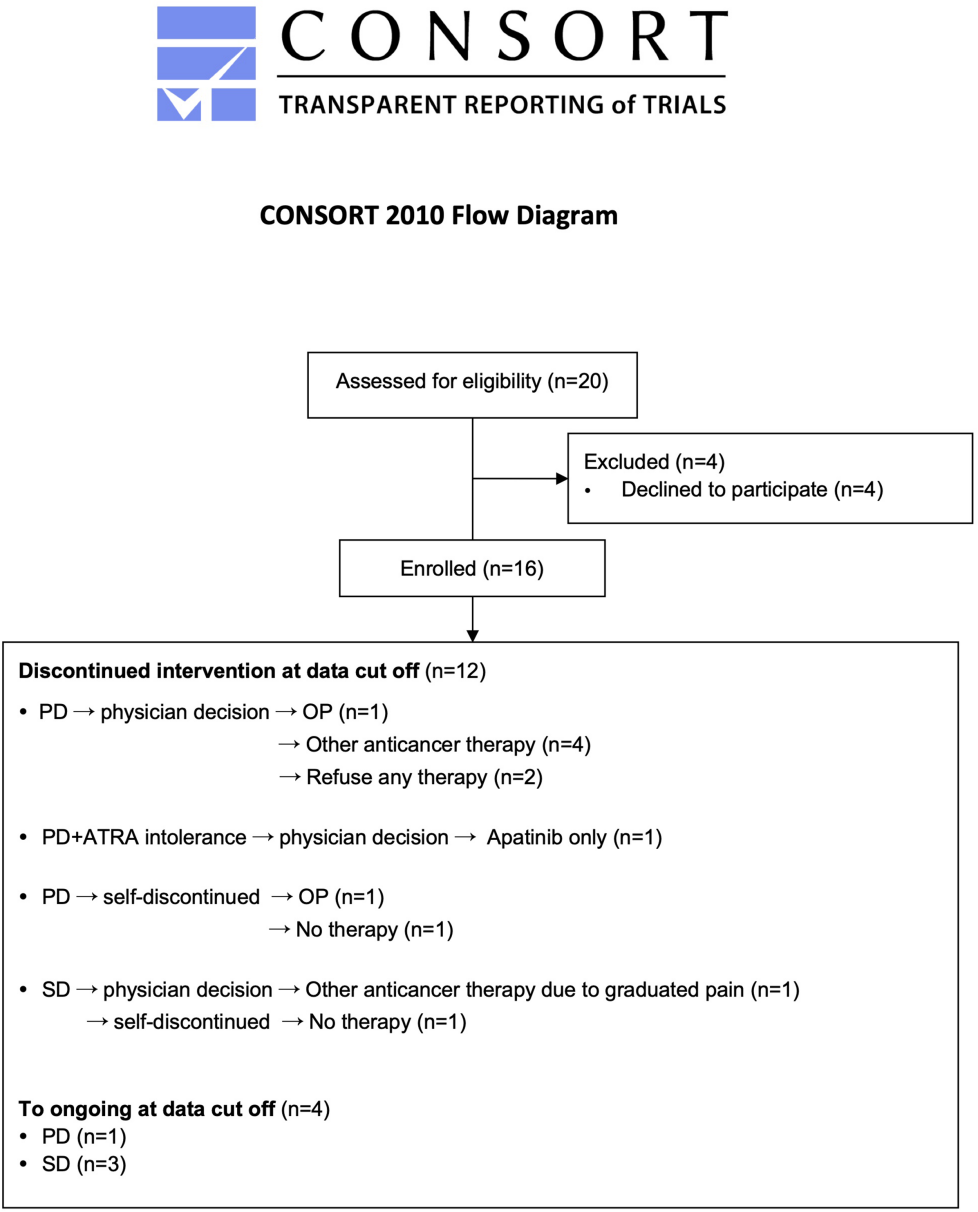
CONSORT flow diagram.

Among all patients of the original phase II study, 16 patients discontinued apatinib due to toxicity intolerance or PD, and furtherly exploratory accepted combination therapy of ATRA (25 mg/m^2^/day) and low‐dose apatinib (250 mg/day) from March 2019 to October 2021. The detailed patients' enrolment was shown in Figure 2.

We included 16 patients (Table 1) with a median age of 53 years (range: 35–69), nine (56.3%) were males and seven (43.8%) were females. All patients received radical surgery and radiotherapy for primary lesion previously. Before receiving the combined therapy, 12 (75%) prior received apatinib as the first‐line therapy and four (25%) as the second‐line therapy. The median of prior apatinib treatment cycles was 6 months (range: 1–12 months). The primary reason for patients turning to combined therapy was disease progression (*n* = 12), among them, three were also caused by intolerant adverse reactions, and other four patients were due to intolerance of apatinib adverse reactions only. We detailed the individual characteristics of patients' prior treatment in Table 1.

**TABLE 1:** Characteristics of 16 patients before receiving combination therapy of apatinib and ATRA.

Pt no	Age/sex	Location of the primary	Stage	Therapy line	Previous treatment cycles of apatinib	Reason for using ATRA
1	59/F	Oropharyngeal wall	Local recurrence and metastasis (lung, liver)	2	6	AEs
2	58/M	Submandibular gland	Metastasis (lung)	2	6	Progression
3	69/M	Sublingual gland	Metastasis (lung)	2	6	Progression
4	50/M	Parotid gland	Metastasis (lung)	2	6	Progression
5	51/F	Sphenoid sinus	Metastasis (lung)	2	6	Progression
6	54/F	Hard palate	Local recurrence and metastasis (lung)	2	4	AEs
7	41/M	Maxillary sinus	Metastasis (lung)	2	6	AEs
8	39/F	Lateral thyroid	Metastasis (lung)	3^a^	4	Progression
9	35/M	Soft palate	Local recurrence and metastasis (lung)	3^b^	9	Progression, AEs
10	41/M	Gingivus	Local recurrence	3^b^	12	Progression, AEs
11	64/F	External auditory canal	Local recurrence	2	10	Progression, AEs
12	58/F	Parotid gland	Metastasis (lung)	2	10	Progression
13	46/F	Root of tongue	Metastasis (lung)	2	10	AEs
14	35/M	Submandibular gland	Metastasis (lung, liver, mediastinum)	3^c^	1	Progression
15	54/M	Maxillary sinus	Local recurrence and metastasis (lung)	2	1	Progression
16	57/M	Maxillary sinus	Metastasis (lung)	2	1	Progression

^a^ First‐line TPF Chemotherapy (Docetaxel + Cisplatin + Fluorouracil).

^b^ First‐line TP Chemotherapy (Paclitaxel + Cisplatin).

^c^ 1st line TP/GP Chemotherapy (Paclitaxel + Cisplatin/Gemcitabine + Cisplatin).

### 3.2 Efficacy

**FIGURE 3:**
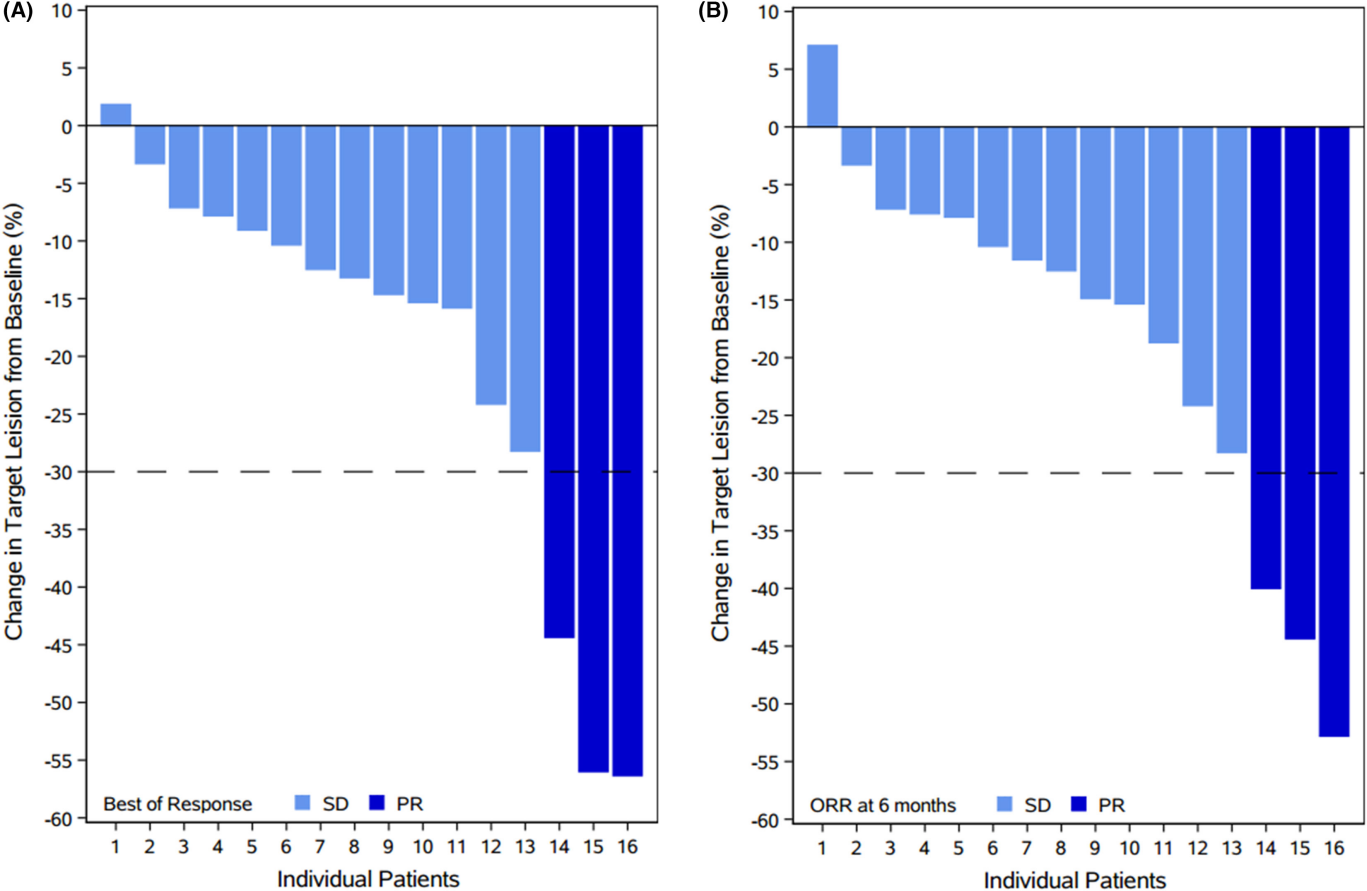
Changes in target lesion from baseline according to the best of response (A) and objective response at 6 months (B).

**FIGURE 4:**
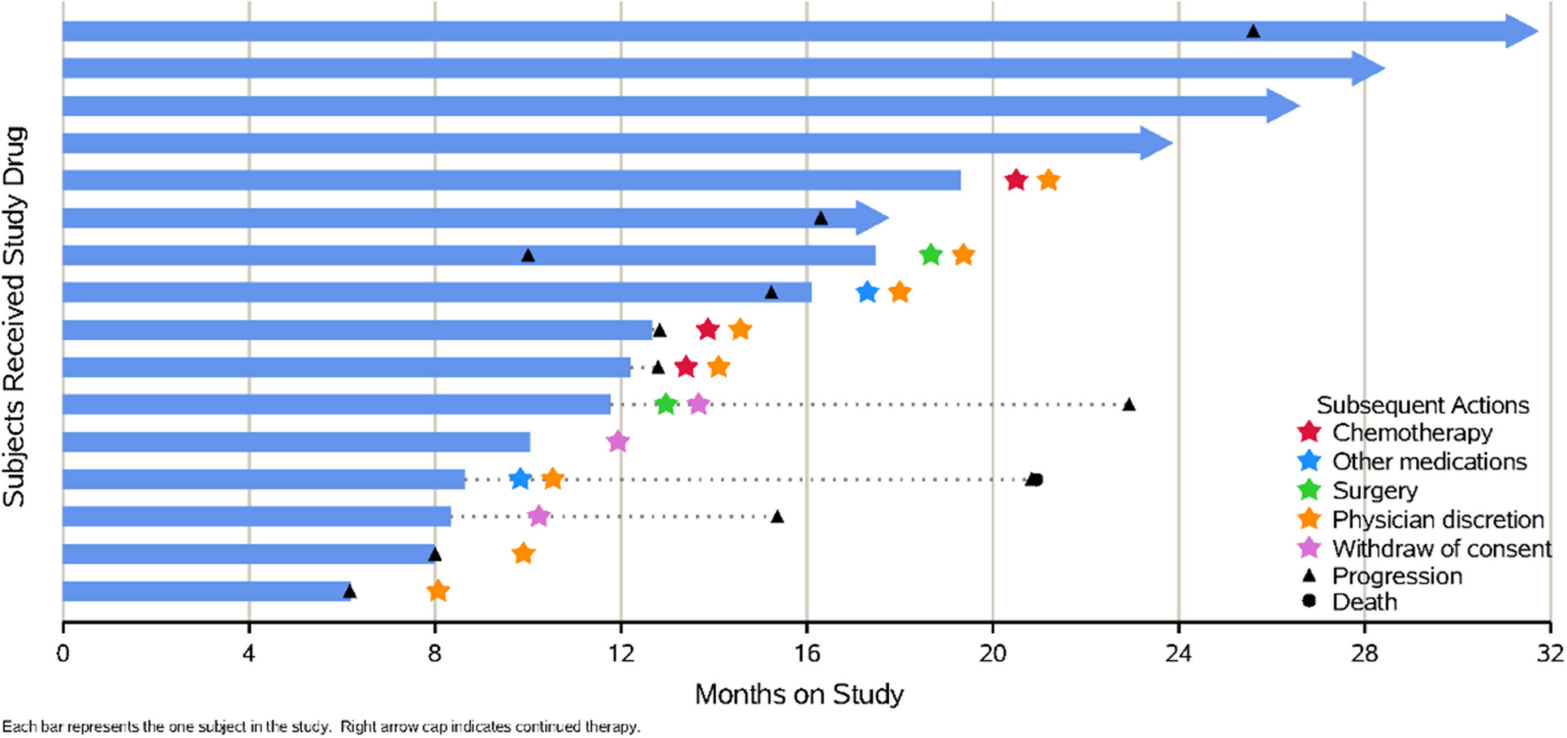
Responses of 16 patients after combination treatment of ATRA and apatinib by October 15, 2021.

**FIGURE 5:**
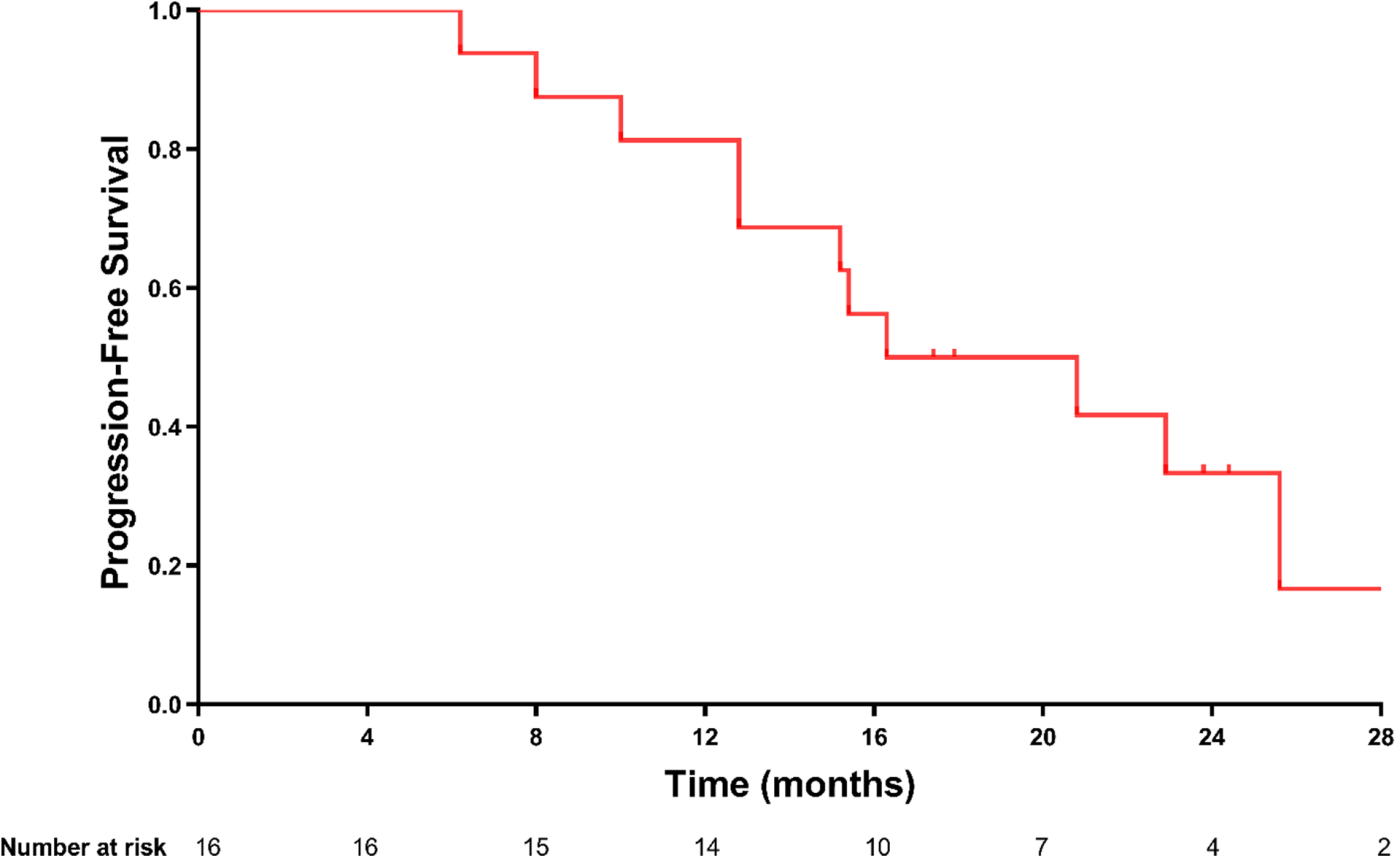
Kaplan–Meier curve for progression‐free survival of 16 patients receiving combination therapy of ATRA and apatinib.

Of 16 patients, three (19%) achieved PR and 13 (81%) achieved SD, with best ORR of 19% and DCR of 100%, respectively. The median time to best ORR was 3.4 months (range:0.2–6.5 months). Among the 13 patients with SD, 12 (92.3%) showed tumor shrinkage (3–28%) and one (7.7%) showed minor tumor enlargement (2%) (Table 2 & Figure 3A). By the 6 months, the ORR and DCR were maintained (Table 2 & Figure 3B). By October 15, 2021, during a median follow‐up duration of 23.9 months (range: 17.8–31.7 months), 11 (68.8%) patients were PD and among them one patient was further died (survival months: 20.9) (Table 2 & Figure 4). The median PFS was 16.3 months (95% CI: 7.2–25.4 months) from initiation of combination therapy, and the 6‐month, 12‐month, and 24‐month PFS rates were 100%, 81.3%, and 33.3%, respectively (Figure 5).

**TABLE 2:** Effectiveness of 16 patients receiving combination therapy of ATRA and apatinib.

Pt No	Best response	Best response time (month)	6 month response	Period of ATRA + apatinib (months)^a^	Time and efficacy of last evaluation (months, response)	ATRA + apatinib discontinuation reason	Follow‐up treatment	Follow‐up (month)^a^
14	PR/56%	2.5	PR/53%	17.5	10.0/PD	Doctor advice^c^ Progression of liver metastases, interventional therapy is recommended to control liver lesions	Liver interventional surgery, the lesion was stable and follow‐up closely	22.2
11	PR/56%	2.3	PR/40%	6.2	6.2/PD	Doctor advice^c^ The local lesion progressed. Considering the lesion was close to the skin and may rupture, antiangiogenic targeted therapy was not suitable to use, chemotherapy was recommended but the patient refused.	None	23.8
16	PR/44%	4.9	PR/44%	10.0	17.4/SD	Self‐discontinuation Patient was unwilling to continue treatment and failed to use other treatment	None	17.9
15	SD/28%	3.5	SD/28%	8.0	16.3/PD	ATRA intolerance Physician suggested change drugs, but patients wanted to continue with apatinib and stop ATRA	Only low‐dose apatinib	17.8
1	SD/24%	6.5	SD/24%	Continue	25.6/PD	—— (The lesion progressed slowly, recommended to switch to use other targeted drugs. Patient want to continue use the drug, considering convenient and tolerable of current regimen)	——	31.7
9	SD/16%	5.6	SD/15%	Continue	17.9/SD	——	——	23.9
7	SD/15%	3.3	SD/15%	8.0	8.0/PD	Doctor advice^c^ Progressed in lung. Physician suggested switching to other targeted drugs, but refused by patient.	None	25.8
3	SD/15%	0.2	SD/19%	Continue	28.4/SD	——	——	28.4
8	SD/13%	1.2	SD/12%	11.8	22.9/PD	Self‐discontinuation Patient thought that the drug was ineffective, so selected surgical treatment although physician did not recommend surgery because of the multiple lesions in lung	surgery	24.4
13	SD/12%	4.2	SD/12%	8.3	15.4/PD	Self‐discontinuation Patient refused to continue current treatment and other treatments after progression.	None	22.5
12	SD/10%	5.6	SD/10%	16.1	15.2/PD	Doctor advice^c^ Progression in lung, other targeted drugs were recommended.	Other targeted drugs	22.8
10	SD/9%	2.3	SD/8%	19.3	23.8/SD	Doctor advice^c^ Aggravation of local pain, chemotherapy was recommended.	Chemotherapy	23.8
6	SD/8%	4.1	SD/8%	Continue	24.4/SD	——	——	26.6
5	SD/7%	4.1	SD/7%	12.2	12.8/PD	Doctor advice^c^ Rapid progression in lung, chemotherapy was recommended.	Chemotherapy	26.9
2^b^	SD/3%	3.3	SD/3%	8.6	20.8/PD	Doctor advice^c^ Progression in lung, other targeted drugs were recommended.	Other targeted drugs	20.9
4	SD/−2%	2.0	SD/−7%	12.7	12.8/PD	Doctor advice^c^ Rapid lung progression, chemotherapy was recommended.	Chemotherapy	28.4

Abbreviations: PR, partial response; SD, stable disease.

^a^ The cut‐off time:October 15, 2021.

^b^ The patient died for the disease progressed.

^c^ The criteria for treatment adjustment and withdraw are as following: 1. Discontinued treatment due to grade 3 apatinib‐related toxic reactions, and then resumed after recovery to grade 2 or below; 2. If grade 3 reaction occurs again, suspending the drug. After and recovering to grade 2 or below, the dose should be halved; 3. If grade 3 reaction still occurs after lowering the dosage, withdrawal should be considered; 4. The patients asked for withdraw due intolerable or unwilling to take medicine; 5. The doctor considers that it is not appropriate to continue treatment.

During the treatment period, 12 patients (75%) finally discontinued the combination therapy for doctor advice or self‐discontinuation. Among them, nine progressed after 6.2–19.3 months of treatment, five of them finally discontinued treatment by doctors' advice, and three stopped by themselves. Among them, two were switched to other types of anti‐angiogenic drugs, two were switched to chemotherapy, and the others did not accept treatment. One patient was evaluated for PD in 25.6 month, but continued the combination therapy without discontinuing the medication, considering the drug accessibility and prior efficacy.

### 3.3 Safety

By October 15, 2021, the 16 patients treated with the combination therapy ranged from 6.2 to 31.7 months, and the combination therapy was well tolerated. The major complications were hyperlipidemia in 10 patients (62.5%), dizziness in nine (56.3%), liver damage in seven (43.75%), and headache in six (37.5%), and all these complications were grade 1 or 2 AEs. Only three (18.75%) patients occurred grade 3 apatinib‐related AEs which were hand‐foot syndrome or albuminuria, and two patients suspended combination therapy due to AEs for 1 week, and then continued but had a dosage reduction (Table 3).

**TABLE 3:** Adverse events of 16 patients receiving combination therapy of ATRA and apatinib.

Pt	Adverse events	Apatinib adjustment	ATRA adjustment
1	Hand‐foot syndrome^b^, dizziness, hyperlipidemia, hemorrhage, angular cheilitis	Discontinuation for 1 week because of grade 3 AE, reused later, used 250 mg Qod	None
2	Albuminuria^b^, dizziness, headache	Discontinuation for 1 week because of grade 3 AE, and then reused for 125 mg bid	None
3	Dizziness, hyperlipidemia, liver damage, hand‐foot syndrome	Dosage reduction and use the two drugs alternately, 250 mg qod	Dosage reduction and use the two drugs alternately, 25 mg/m^2^ qod
4	Liver damage^a^, dizziness, headache, hyperlipidemia	Dosage reduction	None
5	None	Dosage reduction	None
6	Albuminuria^b^, hand‐foot syndrome^a^, Liver damage, angular cheilitis, hyperlipidemia	Dosage reduction and use the two drugs alternately, 250 mg qod	Dosage reduction and use the two drugs alternately, 25 mg/m^2^ qod
7	Liver damage	None	None
8	Albuminuria^a^, dizziness, headache, hyperlipidemia	Irregular medication uses and use the two drugs alternately, 250 mg qod	Irregular medication use and use the two drugs alternately, 25 mg/m^2^ qod
9	None	Reduction, recovery after self‐discontinuation, and then intermittent medication	None
10	Hyperlipidemia, hemorrhage	Discontinuation, and then dosage reduction, and then intermittent medication	None
11	Liver damage^a^, hyperlipidemia	None	None
12	None	None	None
13	Dizziness, headache, hyperlipidemia, angular cheilitis, albuminuria	Dosage reduction	None
14	Liver damaged^a^, dizziness, headache, hyperlipidemia	None	None
15	Dizziness^a^, headache^a^, hemorrhage^a^, hand‐foot syndrome^a^, hyperlipidemia	Discontinuation, and then dosage reduction	Stopped ATRA after 8 cycles for intolerance
16	Dizziness, liver damage, hemorrhage	Discontinuation, dosage reduction, and self‐discontinuation	None

^a^ Grade 2.

^b^ Grade 3; others AE is grade 1.

A total of 12 (75%) patients reduced their dosage of apatinib for AEs. Among them, five patients reduced the dosage of apatinib after suspending the medication, four (25%) patients reduced their dosage of ATRA, and one patient discontinued ATRA after 8 cycles for AE (Table 3).

## 4 DISCUSSION

Treatment options are limited for R/M ACC.^26^ Targeted broad‐spectrum anti‐angiogenesis therapy is a relatively effective treatment method verified for many cancer types. Nevertheless, its associated toxicities or eventual drug resistance limit their use.^27^, ^28^ Lenvatinib and sorafenib are recognized treatments for many types of solid cancers.^29^, ^30^ They have been tried in ACC however showed limited effectiveness.^31^, ^32^, ^33^ Although apatinib monotherapy have been proved to be an effectively antitumor therapy in various cancer types,^34^, ^35^, ^36^ especially for those R/M ACC, its tolerance is still unsatisfactory.^14^

All‐trans retinoic acid serves as an attractive candidate for various malignant tumors.^37^ Through direct dimerization with retinoic acid receptor (RAR) or indirect interaction of downstream metabolites with retinoid X receptor (RXR), ATRA may participate in regulation of many cellular signaling pathways.^38^ The major physiological functions of retinoic acids (RA) have been involved in development, especially in regulation of cell differentiation. It has been suggested that RA regulates the development of pluripotent stem cells,^39^ which plays a critical role in cancer development and metastasis.^40^ Therefore, reducing stemness and promoting cell differentiation may contribute significantly to anti‐cancer activity of ATRA. ATRA have been evaluated in many solid tumors, such as breast cancer, lung cancer, prostate cancer, liver cancer, pancreas cancer, and melanoma, and showed a well efficacy (Table S1). Also, many trial for monotherapy or combination therapy of ATRA have been ongoing (Table S2). For example, ATRA has been suggested to promote cancer cell differentiation in neuroblastoma as well as in acute promyelocytic leukemia.^41^ These functions may enhance the response of tumors to other cytotoxic and targeted therapy by reducing potential escape of tumor cells through dedifferentiation, which is important for both cancer initiation and treatment resistance.^20^, ^21^, ^42^ In this study, addition of ATRA lowers the dose of apatinib and consequently decrease the toxicity, suggesting an increased treatment sensitivity.

This study reviewed 16 patients of R/M ACCHN previously received apatinib monotherapy and then added ATRA therapy. By the last follow‐up date, 12 patients were PD within a range of 17.8–31.7 months of follow‐up. The 6‐month, 12‐month, and 24‐month PFS rates were 100%, 81.3%, and 33.3%, respectively. Comparatively, in the previous study of monotherapy of apatinib with a dosage of 500 mg/day, the 12‐month PFS were 75.2%.^14^ Although both ATRA combined with lower dose apatinib and standard‐dose monotherapy apatinib achieved acceptable efficacy for patients, fewer toxicities and adverse events were observed for the combination therapy. For the standard‐dose of monotherapy apatinib, at least one AE was reported for each patient.^14^ In the present study, a longer duration of tolerable treatment was observed. Therefore, the efficacy and safety of combination therapy of ATRA and low‐dose apatinib in patients with R/M ACCHN is worthy of clinical trials.

Currently, a one‐armed study (NCT03999684) of short‐term ATRA monotherapy (45 mg/m^2^/d) in 18 patients with R/M ACC found the SD rate was 61.1%, however, the treatment duration is relatively short with a median duration of stability of 3.7 months. While our study used ATRA plus apatinib as the salvage therapy for a median of 23.9 months had a 6‐month SD rate of 100% and a 12‐month stability rate of 80%, although there was a dose reduction of 25 mg/m^2^/d. In other words, combination therapy might be effective on ACC and potentially longer than the duration of disease control. Another ongoing trial (NCT04433169) examined ATRA plus apatinib versus monotherapy of apatinib. This is the first study that focused on the efficacy of the combination therapy of ATRA among patients with R/M ACCHN, and the results of this trial will shed a light on the treatment of this entity.

All patients of this study came from a single center and the sample size is a little bit small; and the treatment and severity of the patients might be a large confounder of this study, therefore, further large‐scale well‐designed study are warrant. Also, the retrospective nature might provide several information bias and limitation. On the other hand, the MYB status and pathological types were unclear since the surgical specimens have exceeded the time for further testing. However, this was the first study summarizing the treatment status of patients with R/M ACCHN which provided the critical information on the combination use of ATRA and apatinib. Above shortages will be perfected in the ongoing trial.

All‐trans retinoic acid combined with low‐dose apatinib in patients with R/M ACCHN shows an acceptable efficacy in terms of response and progression‐free survival. Additionally, the reduced‐dose apatinib plus ATRA exhibits an acceptable safety profile, and that the synergistic effects of ATRA plus apatinib may enable reduction in apatinib dose. ATRA combined with apatinib might be a potential treatment option and its efficacy and safety warrant further examined in well‐designed clinical trials. The ongoing clinical trial (NCT04433169) might soon provide more answers regarding the benefits of monotherapy and combination therapy of ATRA for ACC treatment.

## ETHICAL APPROVAL

This study was approved by the ethics committee of Shanghai Ninth People's Hospital affiliated to Shanghai Jiao Tong University, School of Medicine (SH9H‐2020‐T73‐1).

## AUTHOR CONTRIBUTIONS

**Lu‐Lu Ye:** Conceptualization (equal); data curation (equal); formal analysis (equal); writing – original draft (equal); writing – review and editing (equal). **Lin Zhang:** Data curation (equal); writing – review and editing (equal). **Rongrong Li:** Data curation (equal); writing – review and editing (equal). **Xinhua Pan:** Data curation (equal); writing – review and editing (equal). **Jiang Li:** Data curation (equal); writing – review and editing (equal). **Shengjin Dou:** Formal analysis (equal); writing – review and editing (equal). **Wen Jiang:** Formal analysis (equal); writing – review and editing (equal). **Chong Wang:** Formal analysis (equal); writing – review and editing (equal). **Wantao Chen:** Formal analysis (equal); methodology (equal); writing – review and editing (equal). **Guopei Zhu:** Methodology (equal); writing – original draft (equal); writing – review and editing (equal).

## FUNDING INFORMATION

This work is supported by Shanghai Oral and Maxillofacial Tumor Tissue Samples and Bioinformatics Database Professional Technical Service Platform [grant number 21DZ2292000]; and SJTU Trans‐med Awards Research [grant number 20210103].

## CONFLICT OF INTEREST STATEMENT

All authors declare that they have no conflict of interest.

## CLINICAL TRIAL REGISTRATION NUMBER

NCT02775370.

## PATIENT CONSENT STATEMENT

Written informed consent to participate was obtained from participants.

## Supporting information


Table S1.

Table S2.

## Data Availability

The datasets used and/or analyzed during the current study are available from the corresponding author on reasonable request.

## References

[1] Kokemueller H , Eckardt A , Brachvogel P , Hausamen JE . Adenoid cystic carcinoma of the head and neck‐‐a 20 years experience. Int J Oral Maxillofac Surg. 2004;33(1):25‐31. 14690656 10.1054/ijom.2003.0448

[2] Lloyd S , Yu JB , Wilson LD , Decker RH . Determinants and patterns of survival in adenoid cystic carcinoma of the head and neck, including an analysis of adjuvant radiation therapy. Am J Clin Oncol. 2011;34(1):76‐81. 20177363 10.1097/COC.0b013e3181d26d45

[3] Ellington CL , Goodman M , Kono SA , et al. Adenoid cystic carcinoma of the head and neck: incidence and survival trends based on 1973‐2007 surveillance, epidemiology, and end results data. Cancer. 2012;118(18):4444‐4451. 22294420 10.1002/cncr.27408

[4] Dillon PM , Chakraborty S , Moskaluk CA , Joshi PJ , Thomas CY . Adenoid cystic carcinoma: a review of recent advances, molecular targets, and clinical trials. Head Neck. 2016;38(4):620‐627. 25487882 10.1002/hed.23925 PMC6166139

[5] Bradley PJ . Adenoid cystic carcinoma of the head and neck: a review. Curr Opin Otolaryngol Head Neck Surg. 2004;12(2):127‐132. 15167050 10.1097/00020840-200404000-00013

[6] Bobbio A , Copelli C , Ampollini L , et al. Lung metastasis resection of adenoid cystic carcinoma of salivary glands. Eur J Cardiothorac Surg. 2008;33(5):790‐793. 18343149 10.1016/j.ejcts.2007.12.057

[7] Dodd RL , Slevin NJ . Salivary gland adenoid cystic carcinoma: a review of chemotherapy and molecular therapies. Oral Oncol. 2006;42(8):759‐769. 16757203 10.1016/j.oraloncology.2006.01.001

[8] Nightingale J , Lum B , Ladwa R , Simpson F , Panizza B . Adenoid cystic carcinoma: a review of clinical features, treatment targets and advances in improving the immune response to monoclonal antibody therapy. Biochim Biophys Acta Rev Cancer. 2021;1875(2):188523. 33600823 10.1016/j.bbcan.2021.188523

[9] Hao L , Xiao‐lin N , Qi C , Yi‐ping Y , Jia‐quan L , Yan‐ning L . Nerve growth factor and vascular endothelial growth factor: retrospective analysis of 63 patients with salivary adenoid cystic carcinoma. Int J Oral Sci. 2010;2(1):35‐44. 20690417 10.4248/IJOS10005 PMC3475596

[10] Tian S , Quan H , Xie C , et al. YN968D1 is a novel and selective inhibitor of vascular endothelial growth factor receptor‐2 tyrosine kinase with potent activity in vitro and in vivo. Cancer Sci. 2011;102(7):1374‐1380. 21443688 10.1111/j.1349-7006.2011.01939.x PMC11158267

[11] Li J , Zhao X , Chen L , et al. Safety and pharmacokinetics of novel selective vascular endothelial growth factor receptor‐2 inhibitor YN968D1 in patients with advanced malignancies. BMC Cancer. 2010;10:529. 20923544 10.1186/1471-2407-10-529 PMC2984425

[12] Scott AJ , Messersmith WA , Jimeno A . Apatinib: a promising oral antiangiogenic agent in the treatment of multiple solid tumors. Drugs Today (Barc). 2015;51(4):223‐229. 26020064 10.1358/dot.2015.51.4.2320599

[13] Zhang H . Apatinib for molecular targeted therapy in tumor. Drug des Devel Ther. 2015;9:6075‐6081. 10.2147/DDDT.S97235 PMC4654530 26622168

[14] Zhu G , Zhang L , Dou S , et al. Apatinib in patients with recurrent or metastatic adenoid cystic carcinoma of the head and neck: a single‐arm, phase II prospective study. Ther Adv Med Oncol. 2021;13:17588359211013626. 33995600 10.1177/17588359211013626 PMC8111556

[15] Tang XH , Gudas LJ . Retinoids, retinoic acid receptors, and cancer. Annu Rev Pathol. 2011;6:345‐364. 21073338 10.1146/annurev-pathol-011110-130303

[16] Kocher HM , Basu B , Froeling FEM , et al. Phase I clinical trial repurposing all‐trans retinoic acid as a stromal targeting agent for pancreatic cancer. Nat Commun. 2020;11(1):4841. 32973176 10.1038/s41467-020-18636-w PMC7518421

[17] Ni X , Hu G , Cai X . The success and the challenge of all‐trans retinoic acid in the treatment of cancer. Crit Rev Food Sci Nutr. 2019;59(sup1):S71‐S80. 30277803 10.1080/10408398.2018.1509201

[18] Pei J , Flieder DB , Patchefsky A , et al. Detecting MYB and MYBL1 fusion genes in tracheobronchial adenoid cystic carcinoma by targeted RNA‐sequencing. Mod Pathol. 2019;32(10):1416‐1420. 31028361 10.1038/s41379-019-0277-x PMC6763356

[19] Yusenko MV , Trentmann A , Andersson MK , et al. Monensin, a novel potent MYB inhibitor, suppresses proliferation of acute myeloid leukemia and adenoid cystic carcinoma cells. Cancer Lett. 2020;479:61‐70. 32014461 10.1016/j.canlet.2020.01.039

[20] Mandelbaum J , Shestopalov IA , Henderson RE , et al. Zebrafish blastomere screen identifies retinoic acid suppression of MYB in adenoid cystic carcinoma. J Exp Med. 2018;215(10):2673‐2685. 30209067 10.1084/jem.20180939 PMC6170170

[21] Sun B , Wang Y , Sun J , et al. Establishment of patient‐derived xenograft models of adenoid cystic carcinoma to assess pre‐clinical efficacy of combination therapy of a PI3K inhibitor and retinoic acid. Am J Cancer Res. 2021;11(3):773‐792. 33791153 PMC7994170

[22] Hanna GJ , ONeill A , Cutler JM , et al. A phase II trial of all‐trans retinoic acid (ATRA) in advanced adenoid cystic carcinoma. Oral Oncol. 2021;119:105366. 34091189 10.1016/j.oraloncology.2021.105366

[23] Linxweiler M , Kuo F , Katabi N , et al. The immune microenvironment and neoantigen landscape of aggressive salivary gland carcinomas differ by subtype. Clin Cancer Res. 2020;26(12):2859‐2870. 32060100 10.1158/1078-0432.CCR-19-3758 PMC7918996

[24] Bauer R , Udonta F , Wroblewski M , et al. Blockade of myeloid‐derived suppressor cell expansion with all‐trans retinoic acid increases the efficacy of antiangiogenic therapy. Cancer Res. 2018;78(12):3220‐3232. 29674477 10.1158/0008-5472.CAN-17-3415

[25] McCarter M , Tobin RP , Cogswell DT , et al. Pembrolizumab and all‐trans retinoic acid combination treatment of advanced melanoma. J Clin Oncol. 2021;39(15_suppl):9536.

[26] NCCN Clinical Practice Guidelines in Oncology (NCCN Guidelines) . Head and Neck Cancers. National Comprehensive Cancer Network; 2021.

[27] Abdalla AME , Xiao L , Ullah MW , Yu M , Ouyang C , Yang G . Current challenges of cancer anti‐angiogenic therapy and the promise of nanotherapeutics. Theranostics. 2018;8(2):533‐548. 29290825 10.7150/thno.21674 PMC5743565

[28] Montemagno C , Pages G . Resistance to anti‐angiogenic therapies: a mechanism depending on the time of exposure to the drugs. Front Cell Dev Biol. 2020;8:584. 32775327 10.3389/fcell.2020.00584 PMC7381352

[29] Hao Z , Wang P . Lenvatinib in management of solid tumors. Oncologist. 2020;25(2):e302‐e310. 32043789 10.1634/theoncologist.2019-0407 PMC7011622

[30] Guanghan F , Xuyong W , Xiao X . Is the era of sorafenib over? A review of the literature. Ther Adv Med Oncol. 2020;12:1758835920927602. 32518599 10.1177/1758835920927602 PMC7252361

[31] Tchekmedyian V , Sherman EJ , Dunn L , et al. Phase II study of Lenvatinib in patients with progressive, recurrent or metastatic adenoid cystic carcinoma. J Clin Oncol. 2019;37(18):1529‐1537. 30939095 10.1200/JCO.18.01859 PMC6599407

[32] Locati LD , Galbiati D , Calareso G , et al. Patients with adenoid cystic carcinomas of the salivary glands treated with lenvatinib: activity and quality of life. Cancer. 2020;126(9):1888‐1894. 32031693 10.1002/cncr.32754

[33] Thomson DJ , Silva P , Denton K , et al. Phase II trial of sorafenib in advanced salivary adenoid cystic carcinoma of the head and neck. Head Neck. 2015;37(2):182‐187. 24346857 10.1002/hed.23577

[34] Li L , Kong F , Zhang L , et al. Apatinib, a novel VEGFR‐2 tyrosine kinase inhibitor, for relapsed and refractory nasopharyngeal carcinoma: data from an open‐label, single‐arm, exploratory study. Invest New Drugs. 2020;38(6):1847‐1853. 32363427 10.1007/s10637-020-00925-2 PMC7575486

[35] Wang F , Yuan X , Jia J , et al. Apatinib monotherapy for chemotherapy‐refractory metastatic colorectal cancer: a multi‐centre, single‐arm, prospective study. Sci Rep. 2020;10(1):6058. 32269247 10.1038/s41598-020-62961-5 PMC7142071

[36] Sun X , Li J , Li Y , Wang S , Li Q . Apatinib, a novel tyrosine kinase inhibitor, promotes ROS‐dependent apoptosis and autophagy via the Nrf2/HO‐1 pathway in ovarian cancer cells. Oxid Med Cell Longev. 2020;2020:3145182. 32509141 10.1155/2020/3145182 PMC7244982

[37] Giuli MV , Hanieh PN , Giuliani E , et al. Current trends in ATRA delivery for cancer therapy. Pharmaceutics. 2020;12(8):707. 32731612 10.3390/pharmaceutics12080707 PMC7465813

[38] Ghyselinck NB , Duester G . Retinoic acid signaling pathways. Development. 2019;146(13):dev167502. 31273085 10.1242/dev.167502 PMC6633611

[39] Ronn RE , Guibentif C , Moraghebi R , et al. Retinoic acid regulates hematopoietic development from human pluripotent stem cells. Stem Cell Reports. 2015;4(2):269‐281. 25680478 10.1016/j.stemcr.2015.01.009 PMC4325193

[40] Baccelli I , Trumpp A . The evolving concept of cancer and metastasis stem cells. J Cell Biol. 2012;198(3):281‐293. 22869594 10.1083/jcb.201202014 PMC3413352

[41] Bushue N , Wan YJ . Retinoid pathway and cancer therapeutics. Adv Drug Deliv Rev. 2010;62(13):1285‐1298. 20654663 10.1016/j.addr.2010.07.003 PMC2991380

[42] Carvalho J . Cell reversal from a differentiated to a stem‐like state at cancer initiation. Front Oncol. 2020;10:541. 32351900 10.3389/fonc.2020.00541 PMC7174973

