# Supplementary material for: Combined all‐trans retinoic acid with low‐dose apatinib in treatment of recurrent/metastatic head and neck adenoid cystic carcinoma: A single‐center, secondary analysis of a phase II study
Source: Cancer Med. 2023 Feb 3;12(8):9144–55. doi: 10.1002/cam4.5653 (PMC10166967; doi:10.1002/cam4.5653)
Supplement: Supplementary file 1 — Table S1. [file CAM4-12-9144-s002.docx]

Table S1. Recent published trials of ATRA in solid tumors.

| Researcher/Center (Time) | Diseases | Therapeutic | Results | Adverse events |
| --- | --- | --- | --- | --- |
| Oscar (2010) [1] | Advanced non-small cell lung cancer | Control: placebo + cisplatin + paclitaxel  Treatment: ATRA (20mg/m2/day，continuous for 7 days)+ cisplatin + paclitaxel for 2 cycles | Control vs. Treatment group  ORR：25.4% vs 55.8%  PFS：6.0months vs. 8.9 months | 10% patients presented hypertriglyceridemia in treatment group; treatment related toxic effects in two groups: leukopenia, anemia, thrombocytopenia, dermatologic, nausea or vomiting, renal dysfunction, neuropathy, anorexia |
| Bryan (2011) [2] | Recurrent or metastatic breast cancer | ATRA (45mg/m^2^/d，PO daily for 4 days)+paclitaxel (80mg/m^2^/week，IV administered weekly for 3 weeks), repeated in 28 day cycles until disease progression or until no longer tolerated | PR: 17.6%  SD: 58.8%  ORR: 76.4% | Grade 1 or 2 adverse events: alopecia, constipation, fatigue, edema, leukopenia/neutropenia, and neuropathy; grade 3 treatment related adverse events: 1 case for anemia, 2 for nausea and vomiting, 2 pain, and 1 for ataxia; 1 serious adverse event: seizure |
| Kocher (2020) [3] | Locally advanced or metastatic pancreatic cancer | ATRA (45 mg/m^2^/d, bid) + gemcitabine+nab-paclitaxel，28 days for 1 cycle | mPFS: 6.4 months  mOS: 10.9 months | Not reported |
| Hanna (2021) [4] | Recurrent or metastatic adenoid cystic cancer | Cohort 1: ATRA (45mg/m^2^/d, bid, split oral daily dosing on days 1-14 of a 28-day cycle  Cohort 2: if the low efficacy in the first stage of CH1, continuous daily dosing ATRA without interruption during a 28-day cycle (45mg/m^2^/d, bid, for 28 days) | SD: 61%  Median duration of stability：3.7 months  mPFS: 3.2 months | The most commonly reported grade 1-2 toxicities: dry skin and headache；17% of patients with increased cholesterol levels；11%experienced fatigue, nausea, skin rash, and orofacial pain. |
| Shuqun Cheng (2022) [5] | Primary liver cancer with extrahepatic metastasis | Control: FOLFOX, 6 treatment cycles  Treatment: ATRA (20mg, tid) + FOLFOX, 6 treatment cycles | Control vs. treatment  mOS: 9.6 months vs. 14.3 months  mPFS: 1.8 months vs. 3.6 months  CR: 0 vs. 7.0%  DCR: 29.6% vs. 52.6% | Most common adverse events: fatigue, anorexia, nausea, and leukocytopenia; Grade 1 or 2 headaches occurred in approximately 10% of patients in the ATRA + FOLFOX4 group, one patient in each group had liver function damage with NCI CTCAE Grade 3 |
| Richard P. Tobin (2022) [6] | Stage IV melanoma | orally ATRA（150mg/m^2^, totally 12 days treatment with ATRA, for 3 days surrounding each of the first four infusions of pembrolizumab (days -1, 0, and +1) | OR: 71%  mPFS: 20.3 months  CR: 50%  1-year survival rate: 80% | The most common adverse events related with ATRA: headaches, nausea and fatigue |

[1] Arrieta O. Randomized phase II trial of all-trans-retinoic acid with chemotherapy based on paclitaxel and cisplatin as first-line treatment in patients with advanced non-small-cell lung cancer. J Clin Oncol, 2010; 28(21): 3463-71.

[2] Bryan M, Pulte ED, Toomey KC. A pilot phase II trial of all-trans retinoic acid (Vesanoid) and paclitaxel (Taxol) in patients with recurrent or metastatic breast cancer. Invest New Drugs, 2011; 29(6): 1482-7.

[3] Kocher HM, Basu B, Froeling FEM, et al. Phase I clinical trial repurposing all-trans retinoic acid as a stromal targeting agent for pancreatic cancer. Nature communications, 2020; https://doi.org/10.1038/s41467-020-18636-w.

[4] Hannaa GJ, ONeillb A, Cutlera JM, et al. A phase II trial of all-trans retinoic acid (ATRA) in advanced adenoid cystic carcinoma. Oral Oncology, 2021; 119. https://doi.org/10.1016/j.oraloncology.2021.105366

[5] Sun JX, Liu C, Shi J, et al. A novel chemotherapy strategy for advanced hepatocellular carcinoma: a multicenter retrospective study. CMJ, 2021; DOI: 10.1097/CM9.0000000000001952.

[6] Richard PT, Dasha TC, Victoria MC, et al. Targeting MDSC differentiation using ATRA: a phase I/II clinical trial combining pembrolizumab and all-trans retinoic acid for metastatic melanoma. Clin Cancer Res, 2022; https://doi.org/10.1158/1078-0432.CCR-22-2495.
