# Supplementary material for: Combined all‐trans retinoic acid with low‐dose apatinib in treatment of recurrent/metastatic head and neck adenoid cystic carcinoma: A single‐center, secondary analysis of a phase II study
Source: Cancer Med. 2023 Feb 3;12(8):9144–55. doi: 10.1002/cam4.5653 (PMC10166967; doi:10.1002/cam4.5653)
Supplement: Supplementary file 2 — Table S2. [file CAM4-12-9144-s001.docx]

Table S2. Recent ongoing trials of ATRA in solid tumors.

| NCT number | Diseases | Drugs | Study design |
| --- | --- | --- | --- |
| NCT03572387 [1] | Prostate cancer with PSA-only recurrence after local treatment | 1. AZA+ATRA | Treatment: Lupron, 7.5mg/d for 1 month, then using 5-AZA, 40mg/m^2^/d，first day 1-5; ATRA, 45mg/m^2^/d，for the day 3-7, 28 days for 1 cycle, for 3 cycles  Control: Lupron, 7.5mg/d for 1 month |
| NCT04241276 [2] | Grade Ia pancreatic cancer | 1. ATRA+Gemcitabine + nab-paclitaxel | Treatment: ATRA, 45mg/m^2^/d, day 1-5; Gemcitabine, 1000mg/m^2^/d, day 1, 8, 15; nab-paclitaxel, 125mg/m^2^/d, day 1, 8, 15. 28 days of 1 cycle, for 6 cycles of ATRA, using gemcitabine and nab-paclitaxel until disease progression.  Control: Gemcitabine, 1000mg/m^2^/d, day 1, 8, 15; nab-paclitaxel, 125mg/m^2^/d, day 1, 8, 15. 28 days of 1 cycle, treatment until disease progression. |
| NCT05482451 [3] | Chemotherapy-refractory advanced or metastatic pancreatic adenocarcinoma. | Nivolumab+ATRA | Nivolumab, 3mg/kg, day 1; ATRA, 45mg/m2/d, day 1-14. The dose of ATRA was increased to 60 mg/m2 from the second course cycle and 75 mg/m2 from the third course cycle if patients tolerated the treatment.  The treatment cycle will repeat every 2 weeks. |
| NCT04113863 [4] | Operable HR-positive/ HER2-negative early breast cancer | ATRA+ Anastrozole | Treatment: ATRA, 45mg/m^2^/d (two daily administrations of 22.5 mg/m^2^); Anastrozole 1mg/die for 28 days.  Control: Anastrozole 1mg/die for 28 days. |
| NCT04919369 [5] | Recurrent or Metastatic Non-Small Cell Lung Cancer | Atezolizumab+ATRA | Atezolizumab, day 1; ATRA, day 1-3. Cycles repeat every 21 days for 3 cycles. |
| NCT04433169 [6] | Recurrent/Metastatic Adenoid Cystic Carcinoma of the Head and Neck | ATRA | Treatment: ATRA, 20mg tid, for 28 consecutive days, 28 days per cycle (q4w), 6 planned cycles; combined with the treatment regimen chosen by the investigator since Day 6 of cycle 1.  Control: The investigator chooses the treatment regimen based on the following regimens (including but not limited to: 1. VEGFR inhibitor; 2. chemotherapy). |

[1] A Pilot Study of 5-AZA and ATRA for Prostate Cancer with PSA-only Recurrence After Local Treatment. NCT03572387

[2] Phase IIb Randomised Trial of ATRA in a Novel Drug Combination for Pancreatic Cancer (STARPAC2). NCT04241276

[3] Nivolumab and All-trans Retinoic Acid for Pancreatic Cancer. NCT05482451.

[4] ATRA Trial - Activity of ATRA in Combination with Anastrozole in Pre-Operative Phase of Operable HR-Positive/HER2-Negative Early Breast Cancer eBC ATRA Trial (ATRA). NCT04113863

[5] All-Trans Retinoic Acid (ATRA) and Atezolizumab for the Treatment of Recurrent or Metastatic Non-Small Cell Lung Cancer. NCT04919369.

[6] All-trans Retinoic Acid (ATRA) in the Treatment of Recurrent/Metastatic Adenoid Cystic Carcinoma of the Head and Neck (Aplus). NCT04433169.
